# FoxO6-mediated ApoC3 upregulation promotes hepatic steatosis and hyperlipidemia in aged rats fed a high-fat diet

**DOI:** 10.18632/aging.205610

**Published:** 2024-03-03

**Authors:** Dae Hyun Kim, Seulah Lee, Sang Gyun Noh, Jaewon Lee, Hae Young Chung

**Affiliations:** 1 Department of Food Science and Technology, College of Natural Resources and Life Science, Pusan National University, Miryang-si, Gyeongsangnam-do 50463, Republic of Korea; 2 Department of Pharmacy, Research Institute for Drug Development, College of Pharmacy, Pusan National University, Geumjeong-gu, Busan 46241, Republic of Korea

**Keywords:** HFD-feeding aging, forkhead transcription factor O6, ApoC3, lipid accumulation, hepatic steatosis

## Abstract

FoxO6, an identified factor, induces hyperlipidemia and hepatic steatosis during aging by activating hepatic lipoprotein secretion and lipogenesis leading to increased ApoC3 concentrations in the bloodstream. However, the intricate mechanisms underlying hepatic steatosis induced by elevated FoxO6 under hyperglycemic conditions remain intricate and require further elucidation.

In order to delineate the regulatory pathway involving ApoC3 controlled by FoxO6 and its resultant functional impacts, we employed a spectrum of models including liver cell cultures, aged rats subjected to HFD, transgenic mice overexpressing FoxO6 (FoxO6-Tg), and FoxO6 knockout mice (FoxO6-KO).

Our findings indicate that FoxO6 triggered ApoC3-driven lipid accumulation in the livers of aged rats on an HFD and in FoxO6-Tg, consequently leading to hepatic steatosis and hyperglycemia. Conversely, the absence of FoxO6 attenuated the expression of genes involved in lipogenesis, resulting in diminished hepatic lipid accumulation and mitigated hyperlipidemia in murine models. Additionally, the upregulation of FoxO6 due to elevated glucose levels led to increased ApoC3 expression, consequently instigating cellular triglyceride mediated lipid accumulation. The transcriptional activation of FoxO6 induced by both the HFD and high glucose levels resulted in hepatic steatosis by upregulating ApoC3 and genes associated with gluconeogenesis in aged rats and liver cell cultures.

Our conclusions indicate that the upregulation of ApoC3 by FoxO6 promotes the development of hyperlipidemia, hyperglycemia, and hepatic steatosis *in vivo*, and *in vitro*. Taken together, our findings underscore the significance of FoxO6 in driving hyperlipidemia and hepatic steatosis specifically under hyperglycemic states by enhancing the expression of ApoC3 in aged rats.

## INTRODUCTION

Aging is closely linked to non-alcoholic fatty liver disease (NAFLD) and insulin resistance, primarily due to the compromised homeostatic capacity resulting from the growing elderly population [1]. Insulin resistance denotes a cellular state where responses to insulin, crucial for glucose uptake, diminish. Consequently, insulin fails to facilitate glucose uptake, causing hyperglycemia and hyperinsulinemia, prompting increased insulin secretion by pancreatic β-cells to regulate glucose levels. Diabetic individuals produce excessive glucose and triglycerides (TG), contributing to hyperglycemia and hypertriglyceridemia, respectively [2]. The disruption in balancing the production and breakdown of TG-containing lipoproteins, particularly very low-density lipoproteins (VLDLs), leads to hypertriglyceridemia. Age-related NAFLD amplifies mortality risks among the elderly [3]. Given the projected rise in the elderly population, investigating the correlation between hepatic steatosis and aging becomes imperative. Understanding how hyperglycemia triggers age-related hepatic steatosis necessitates further exploration. NAFLD is a liver pathology characterized by the accumulation of TG within hepatocytes and can progress to non-alcoholic steatohepatitis (NASH) [4]. Excessive accumulation of lipids in the liver through the advancement of hepatic steatosis has been associated with hyperglycemia.

The forkhead transcription factor O (FoxO) proteins, including FoxO1/3/4/6 in mammals, constitute an essential group [5]. Insulin signaling induces FoxO inactivation by phosphorylating FoxO, causing its translocation from the nucleus to the cytosol [6–8]. Furthermore, increased levels of reactive oxygen species (ROS) and fatty acids promote FoxO proteins through the JNK signaling pathway [9, 10]. Studies have indicated that constitutively active FoxO1 upregulates the transcription of the lipogenic sterol regulatory element binding protein 1c (SREBP-1c) gene, leading to accumulation of TG in the liver [11]. Another investigation reported FoxO1’s involvement in VLDL production and associated TG in the liver, influencing hypertriglyceridemia by modulating the microsomal TG transfer protein (MTP) in mice [12]. Intriguingly, FoxO1 adjusts fat cell differentiation through the peroxisome proliferator-activated receptor γ (PPARγ) [13] and can bind to the PPARγ promoter, suppressing its transcription [14]. In contrast, FoxO6 notably upregulates PPARγ expression under insulin-resistant conditions in the liver of diabetic db/db mice, consequently stimulating liver lipogenesis and increasing fat content [15]. Hepatic FoxO1, associated with insulin resistance, plays a crucial role in impairing ApoC3 production and contributing to hypertriglyceridemia in obese mice [16]. Additionally, genes encoding ApoA1/C3/A4/A5 have been identified as risk alleles associated with the occurrence of hypertriglyceridemia in humans [17, 18]. Presently, the precise mechanistic relationship between FoxO6 and ApoC3 remains incompletely understood. Additional studies are necessary to elucidate the roles of FoxO6 and ApoC3 in the regulation of liver lipid metabolism, particularly in aged rats fed a high-fat diet (HFD).

Apolipoprotein C3 (ApoC3) is an apolipoprotein found within the spectrum of high-density lipoproteins (HDL), VLDL, and chylomicrons circulating in the bloodstream [19]. Primarily synthesized in the liver, with a smaller production in the intestine, ApoC3 significantly influences TG metabolism through various signaling pathways. Acting as an inhibitor, ApoC3 targets key enzymes like lipoprotein lipase (LPL) and hepatic lipase involved in TG hydrolysis within VLDL and chylomicrons post-absorption stages [20–22]. Increased ApoC3 levels impede liver uptake and the clearance of TG-containing lipoprotein remnants [23, 24]. This regulatory action of ApoC3 on lipoprotein metabolism is mediated by low-density lipoprotein (LDL) receptor-related pathways, independent of LPL levels [25]. In addition to its extracellular functions, ApoC3 also modulates increased VLDL-TG secretion within liver cells [26–29]. Transgenic mice expressing ApoC3 exhibit persistent hypertriglyceridemia from birth, while ApoC3 deficiency in mice leads to enhanced TG hydrolysis and clearance, resulting in reduced circulating TG levels [30–33]. Similarly, individuals with ApoC3 mutations display lowered plasma TG concentrations and reduced risk of coronary artery disease [34, 35]. Studies involving non-human primates and patients with familial chylomicronemia demonstrate that diminishing plasma ApoC3 levels using antisense oligonucleotides significantly decreases plasma TG levels [36, 37]. This collective evidence highlights the connection both ApoC3 and hypertriglyceridemia, positioning ApoC3 as a potential therapeutic mark for managing elevated TG levels [38, 39]. Additionally, ApoC3 expression induces inflammation in endothelial cells [40] and adipose tissue [41]. Elevated ApoC3 levels have been associated with various disorders, including metabolic syndrome [42] and insulin resistance [43, 44] *in vivo*.

In our investigation, we utilized aged rats fed an HFD to explore the relationship between ApoC3 expression and FoxO6. We observed that HFD-induced FoxO6 activity in aged rats led to an increase in hepatic ApoC3 expression. Furthermore, when cells were exposed to high-glucose (HG) treatment, this effect was heightened. The heightened expression of FoxO6 in the liver of aged rats contributed to dysfunctional TG metabolism. Changes in lipids have been associated with various metabolic diseases [45]. Given that the liver primarily functions as a metabolic organ characterized by elevated basal energy consumption and a reliance on fatty acid oxidation as its primary energy source [46, 47], the disruption in fatty acid oxidation has been proposed to drive abnormal TG accumulation, leading to lipotoxicity and the progression of liver diseases. In this context, our investigation aimed to examine the relationship between age-related hepatic steatosis and hyperglycemia, alongside studying how FoxO6 regulates ApoC3 during hyperglycemic conditions in both liver tissues and cells. Our objective is to gain a deeper understanding of the fundamental molecular mechanisms underlying hyperlipidemia in aged rats fed a high-fat diet.

## RESULTS

### Effects of HFD on serum lipids, glucose, and insulin during aging

The standard plasma metabolites were assessed to evaluate the effects of HFD in the context of aging. Previous reports have highlighted changes in body weight and food intake throughout the course of the experiment [48].

**Figure 1:**
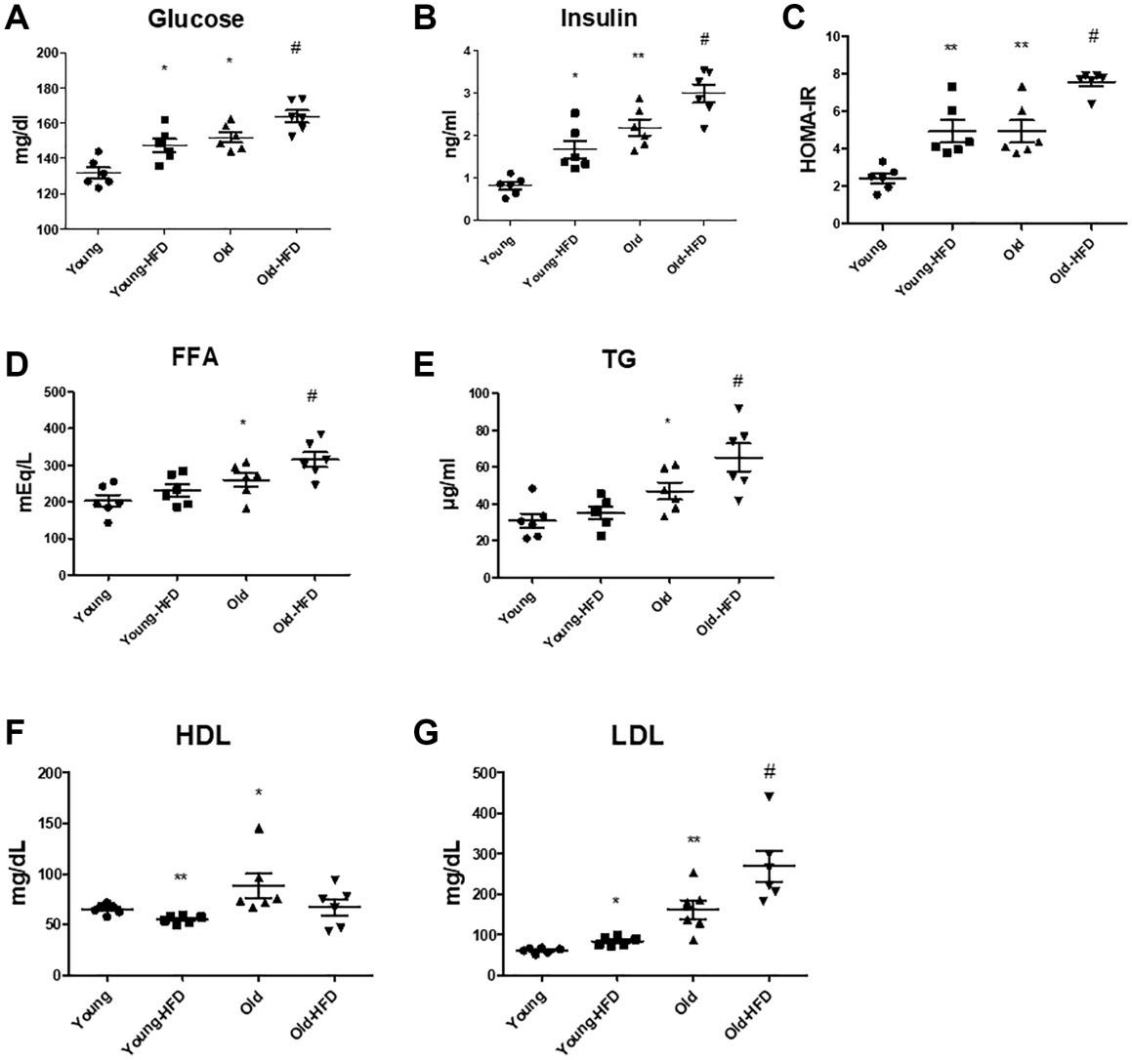
**Aging-related serum changes in insulin resistance and lipogenesis.** (**A**) Glucose levels, (**B**) insulin levels, and (**C**) HOMA-IR scores were determined. (**D**) FFA (free fatty acid), (**E**) TG, (**F**) HDL, and (**G**) LDL levels in the serum of HFD-fed aged rats (each *n* = 6). Results of one-factor ANOVA: ^*^p < 0.05, ^**^p < 0.01 vs. young rats; ^#^*p* < 0.05 vs. old rats.

The levels of glucose and insulin displayed notable increases in the aged groups compared to the younger groups, and notably, these levels were even higher in the aged rats subjected to an HFD compared to the non-HFD aged rats (Figure 1A, 1B). The Homeostatic Model Assessment for Insulin Resistance (HOMA-IR) data depicted in Figure 1C exhibited significantly elevated scores in the HFD-aged rat group compared to the non-HFD aged rats, indicating an exacerbation of HFD-induced insulin resistance through HOMA-IR. However, plasma levels of free fatty acids (FFA) and TG were markedly higher in the aged groups compared to the younger groups, with further significant elevations observed in the HFD-aged groups relative to the non-HFD aged groups (Figure 1D, 1E). In addition, plasma LDL levels were significantly increased in the aged groups when compared to the younger groups, and notably higher in the HFD-aged groups compared to the non-HFD aged groups (Figure 1G). However, HDL levels did not exhibit significant changes in the HFD-fed aged groups compared to the non-HFD aged groups (Figure 1F).

### Effects of FoxO6 on ApoC3-mediated hyperlipidemia in HFD-fed aged rats

**Figure 2:**
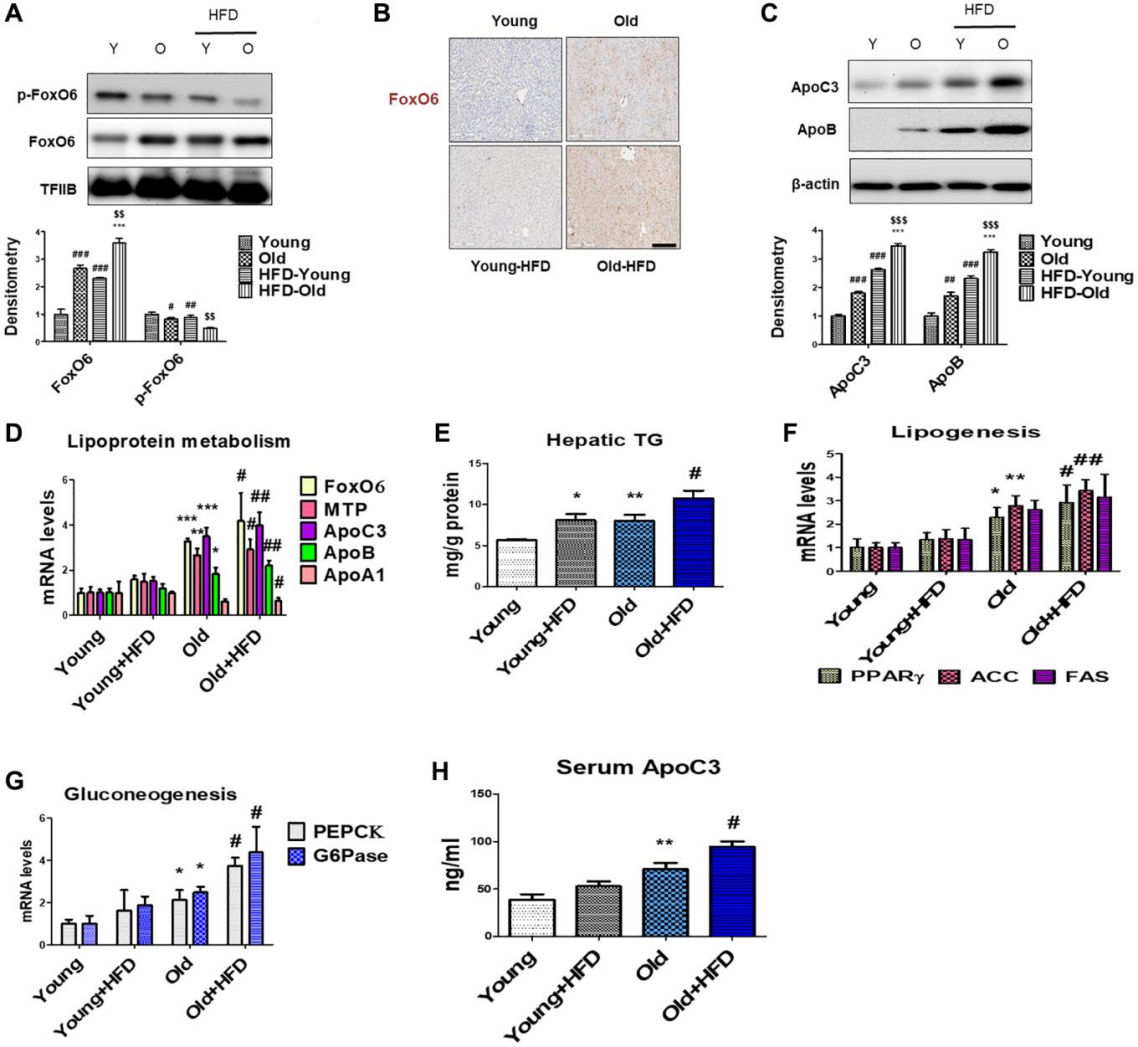
**Aging-related increase in hyperlipidemia through FoxO6-induced apolipoprotein expression.** (**A**) Western blotting was performed to investigate the protein expression levels of p-FoxO6 and FoxO6 in the liver of aged rats. TFIIB was the loading control of the nuclear fractions. Results are representative of three independent experiments. Bars in the densitometry data represent the mean ± S.E., and significance was determined using one-factor ANOVA: ^#^*p* < 0.05, ^##^*p* < 0.01, ^###^*p* < 0.001 vs. Young; ^***^p < 0.001 vs. HFD-Young; ^$$^*p* < 0.01 vs. Old. (**B**) Immunohistochemical staining for FoxO6 in the liver of aged rats. Scale bar: 100 μm. (**C**) Western blotting was performed to investigate the protein levels of ApoC3 and ApoB in the liver of aged rats. β-actin was the loading control of the cytosolic fraction. Bars in the densitometry data represent the mean ± S.E., and significance was determined using one-factor ANOVA: ^##^*p* < 0.01, ^###^*p* < 0.001 vs. Young; ^***^p < 0.001 vs. HFD-Young; ^$$$^*p* < 0.001 vs. Old. (**D**) Real-time PCR analyses were performed to measure the mRNA levels of FoxO6, MTP, ApoC3, ApoB, and ApoA1. Results of one-factor ANOVA: ^*^p < 0.05, ^**^p < 0.01, ^***^p < 0.001 vs. Young; ^#^*p* < 0.05, ^##^*p* < 0.01 vs. Young-HFD. (**E**) Hepatic TG in HFD-fed aged rats. Results of one-factor ANOVA ^*^p < 0.05, ^**^p < 0.01 vs. Young; ^#^*p* < 0.05 vs. Old. (**F**) Real-time PCR analyses were performed for measuring the mRNA levels of PPARγ, ACC, and FAS. Results of one-factor ANOVA: ^*^p < 0.05, ^**^p < 0.01 vs. Young; ^#^*p* < 0.05, ^##^*p* < 0.01 vs. Young-HFD. (**G**) G6Pase and PEPCK mRNA levels (gluconeogenesis-related genes) in the livers of HFD-fed aged rats. Results of one-factor ANOVA: ^*^p < 0.05 vs. Young; ^#^*p* < 0.05, ^##^*p* < 0.01 vs. Young-HFD. (**H**) Plasma levels of ApoC3 were determined. Results of one-factor ANOVA: ^**^p < 0.01 vs. Young; ^#^*p* < 0.05 vs. Young-HFD.

Insulin signaling through Akt primarily inhibits FoxO6 activity. Dephosphorylation at Thr26 and Ser184 sites in FoxO6 enhances its activity, consequently leading to increased levels of hyperglycemia [49]. Our data indicate that there is dephosphorylation of FoxO6 in the livers of aged rats (Figure 2A), while simultaneously observing increased FoxO6 expression levels, as evidenced by immunohistochemical staining (Figure 2B). Genes associated with impaired lipoprotein metabolism such as ApoC3, ApoB, MTP, and ApoA1 were detected in the aged groups fed an HFD. Our investigation aimed to assess the impact of ApoC3 and ApoB during the aging process by examining whether the HFD significantly elevated the levels of ApoC3 and ApoB in aged rats (Figure 2C). Additionally, we assessed lipoprotein metabolism using real-time PCR. As anticipated, the levels of MTP, ApoC3, and ApoB were elevated in aged rats, with further increases observed in the aged rats subjected to the HFD (Figure 2D).

To delineate the influence of an HFD on lipid metabolism associated with aging, we quantified hepatic TG content and assessed the expression of lipogenic genes. Aged livers exhibited elevated TG levels compared to their young counterparts on a normal diet, with a further substantial increase observed in HFD-fed aged livers (Figure 2E). Histological examination via H&E staining demonstrated pronounced lipid droplet accumulation in the livers of aged rats, a phenomenon exacerbated in HFD-fed aged rat livers (Supplementary Figure 1). Concurrently, expression analysis of genes involved in lipogenesis, including PPARγ, ACC, and FAS, revealed significant upregulation in aged rat livers compared to young counterparts, further amplified in both young and aged rats subjected to the HFD (Figure 2F). Additionally, the HFD-mediated aging process corresponded to heightened expression of gluconeogenesis-associated genes, such as PEPCK and G6Pase (Figure 2G). Remarkably, there was a notable increase in plasma ApoC3 levels in HFD-fed aged rats (Figure 2H). These observations underscore an escalation in hepatic triglyceride content and gluconeogenesis during the aging process. Furthermore, HFD-fed aged rats displayed increased vulnerability to lipid accumulation and hyperglycemia, potentially influenced by the activation of FoxO6, alterations in lipoprotein metabolism, and modulation of lipogenesis gene expression.

### Effects of glucose on lipid accumulation in liver cells

In our investigation focusing on liver cells, we delved into insulin signaling since glucose regulation primarily occurs through insulin in the liver. Supplementary Figure 2 illustrates our findings. We observed an upregulation in the Ser307 phosphorylation of IRS1, a crucial substrate protein marker associated with insulin resistance, within these liver cells. Conversely, the phosphorylation levels of Tyr632 in IRS1 and Ser473 in Akt were notably reduced under HG conditions.

**Figure 3:**
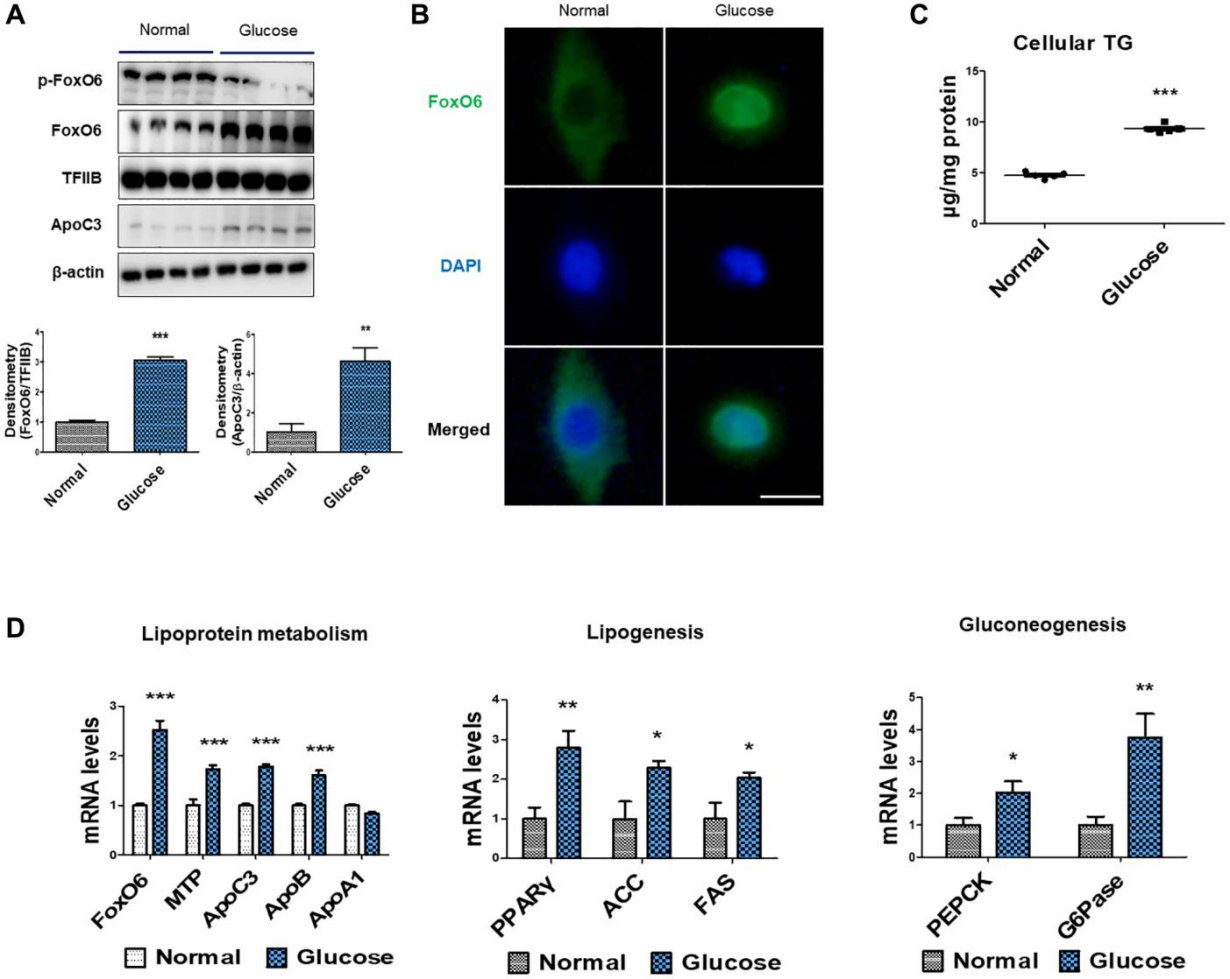
**High-glucose induced hyperlipidemia through FoxO6-mediated ApoC3 expression.** (**A**) Western blot was used to detect p-FoxO6, FoxO6 in nuclear extracts, and ApoC3 in cytoplasmic extracts after treatment of AC2F cells with glucose (25 mM) for 6 h. TFIIB was the loading control of the nuclear fractions, whereas β-actin was the loading control of the cytosolic fractions. Results are representative of three independent experiments. Bars in the densitometry data represent the mean ± S.E., and significance was determined using one-factor ANOVA: ^**^p < 0.01, ^***^p < 0.001 vs. Normal. (**B**) Immunohistochemical staining for FoxO6 in cells with high-glucose treatment. Scale bar: 100 μm. (**C**) Cellular triglyceride contents after glucose treatment (25 mM) for 24 h was measured using a colorimetric assay. Results of one-factor ANOVA: ^***^p < 0.001 vs. non-treated cells. Three independent experiments were performed and similar results were obtained. (**D**) Real-time PCR analyses were conducted for measuring the mRNA levels of the lipoprotein metabolism-related genes (MTP, ApoC3, ApoB, and ApoA1), lipogenesis genes (PPARγ, FAS, and ACC) and gluconeogenesis-related genes (PEPCK and G6Pase). Three independent experiments were performed and similar results were obtained. Results of one-factor ANOVA: ^*^p < 0.05, ^**^p < 0.01, ^***^p < 0.001 vs. non-treated cells.

To explore the connection between hyperglycemia and lipid accumulation, we conducted experiments using serum-starved AC2F cells treated with high glucose concentrations (25 mM). Under these conditions, the treatment with HG notably increased the expression of FoxO6 and ApoC3 (Figure 3A). Additionally, an assessment via immunohistochemical staining revealed that FoxO6 transferred from the nucleus to the cytoplasm in response to HG conditions (Figure 3B). We further investigated the impact of glucose on lipid accumulation within AC2F cells. Notably, there was a significant glucose-dependent rise in cellular TG content (Figure 3C). Moreover, through real-time PCR analysis of lipoprotein metabolism, we observed that MTP, ApoC3, and ApoB levels were elevated under HG conditions. Concurrently, genes associated with lipogenesis (PPARγ, ACC, and FAS) exhibited increased expression in HG-treated cells (Figure 3D). Conversely, there was a notable decrease in the expression levels of genes involved in β-oxidation (PPARα, CPT1α, and ACOX1) in HG-treated cells (Figure 3D). Furthermore, HG exposure correlated with upregulated expression of gluconeogenesis-associated genes (PEPCK and G6Pase) (Figure 3D). Collectively, these findings suggest that high glucose levels promoted lipid accumulation by influencing the expression levels of genes related to gluconeogenesis.

### Increased lipid accumulation because of FoxO6 in AC2F cells

The heightened activity of FoxO has been previously documented in situations where insulin levels decrease [6]. In our investigation, we explored the impact of FoxO6 overexpression on the expression of ApoC3 in AC2F cells. Specifically, cells were subjected to treatment with either a vehicle or the constitutively active form of FoxO6 (FoxO6-CA) at a concentration of 100 MOI (multiplicity of infection).

**Figure 4:**
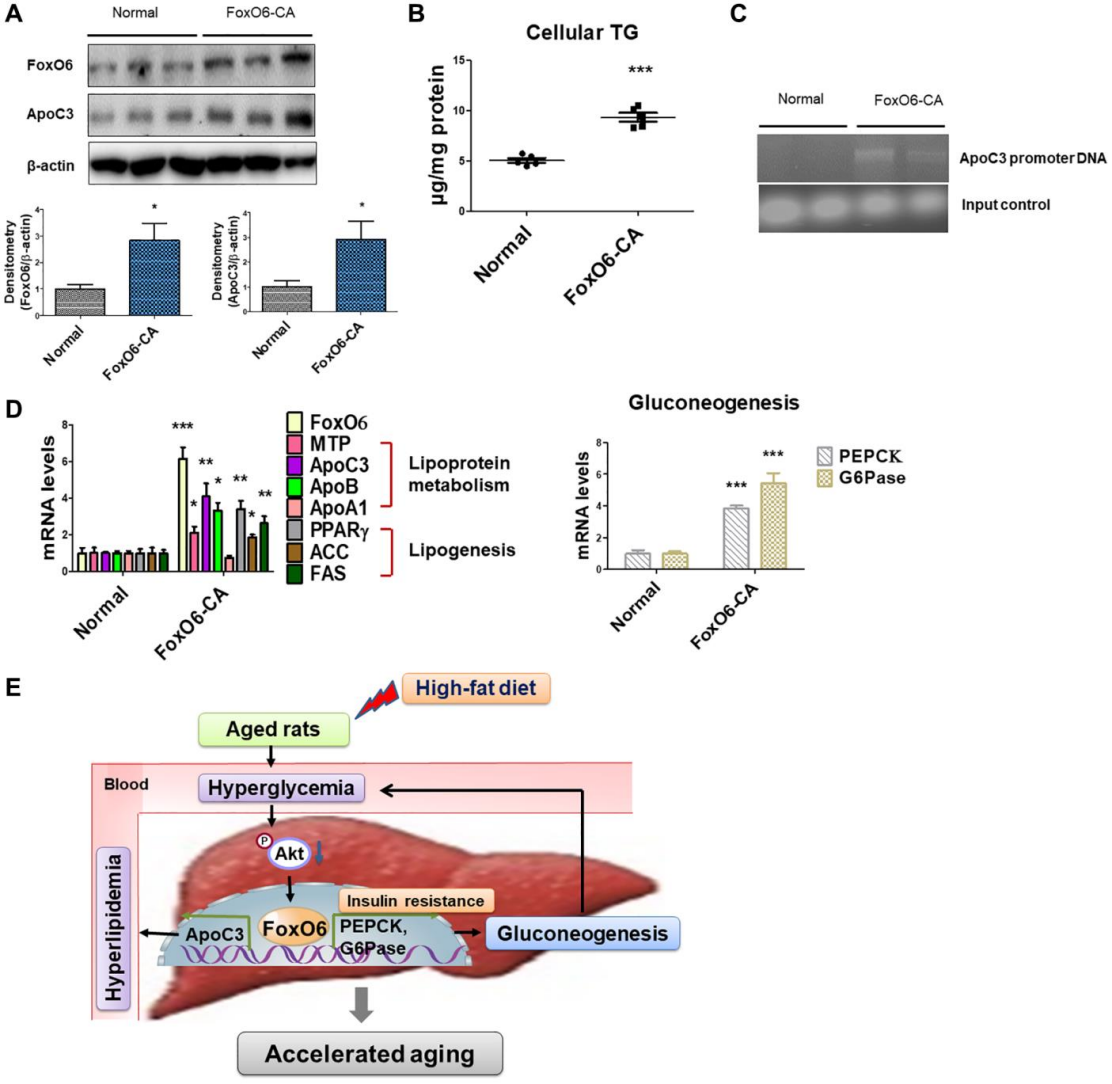
**FoxO6 regulates hyperlipidemia through ApoC3 expression in FoxO6 virus-treated cells.** (**A**) Expression of FoxO6 and ApoC3 by FoxO6. AC2F cells were grown to 80% confluence in 100-mm dishes using DMEM and then stimulated with 100 MOI of FoxO6 for 24 h and analyzed using western blotting using the appropriate antibody. Results are representative of three independent experiments. Bars in densitometry data represent the mean ± S.E., and significance was determined using one-factor ANOVA: ^*^p < 0.05 vs. Normal. (**B**) Cellular TG contents were measured using a colorimetric assay. Results of one-factor ANOVA: ^***^p < 0.001 vs. untreated cells. (**C**) FoxO6 binds to the ApoC3 promoter in liver cells. The cells were subjected to ChIP assay using rabbit pre-immune IgG and an anti-FoxO6 antibody. Immunoprecipitates were subjected to PCR using rat ApoC3 promoter-specific primers. (**D**) Cells incubated without or with FoxO6 (100 MOI) for 24 h were subjected to real-time PCR analyses to determine the mRNA levels of TG synthesis genes (MTP, ApoC3, ApoB, and ApoA1), lipogenesis genes (PPARγ, ACC, and FAS), and gluconeogenesis-related genes (PEPCK and G6Pase), using the β-actin gene as a control. Results of one-factor ANOVA: ^*^p < 0.05, ^**^p < 0.01, ^***^p < 0.001 vs. untreated cells. (**E**) Predicted mechanism in aged liver tissues after HFD administration against lipid accumulation.

The outcomes indicated that treatment with FoxO6-CA led to a notable increase in the expression of ApoC3 (Figure 4A). Additionally, cellular TG levels were elevated in cells treated with FoxO6-CA compared to those in normal cells (Figure 4B). Following a 24-hour treatment period, Chromatin Immunoprecipitation (ChIP) analysis was conducted using FoxO6 antibody or control IgG. The results revealed the presence of FoxO6 in the immunoprecipitated products obtained with the anti-FoxO6 antibody, contrasting with the absence of FoxO6 in products immunoprecipitated using control IgG or in mock-immunoprecipitated products (Figure 4C). Subsequent PCR analysis identified the amplification of a specific DNA fragment (614 bp) corresponding to the nucleotide region (−284/−897 nt) of the ApoC3 promoter within the DNA products co-immunoprecipitated with anti-FoxO6 (Figure 4C). Furthermore, an assessment of lipoprotein metabolism via real-time PCR unveiled increased levels of MTP, ApoC3, and ApoB in cells treated with FoxO6-CA. Similarly, the expression level of fat synthesis genes related to PPAR-γ was heightened in FoxO6-CA treated cells. Additionally, both PEPCK and G6Pase showed increased expression in cells treated with FoxO6-CA (Figure 4D). These findings suggest that the activation of FoxO6 exerted a potent stimulatory effect on lipid accumulation within liver cells.

### Deletion of ApoC3 suppressed lipid accumulation *in vitro*

**Figure 5:**
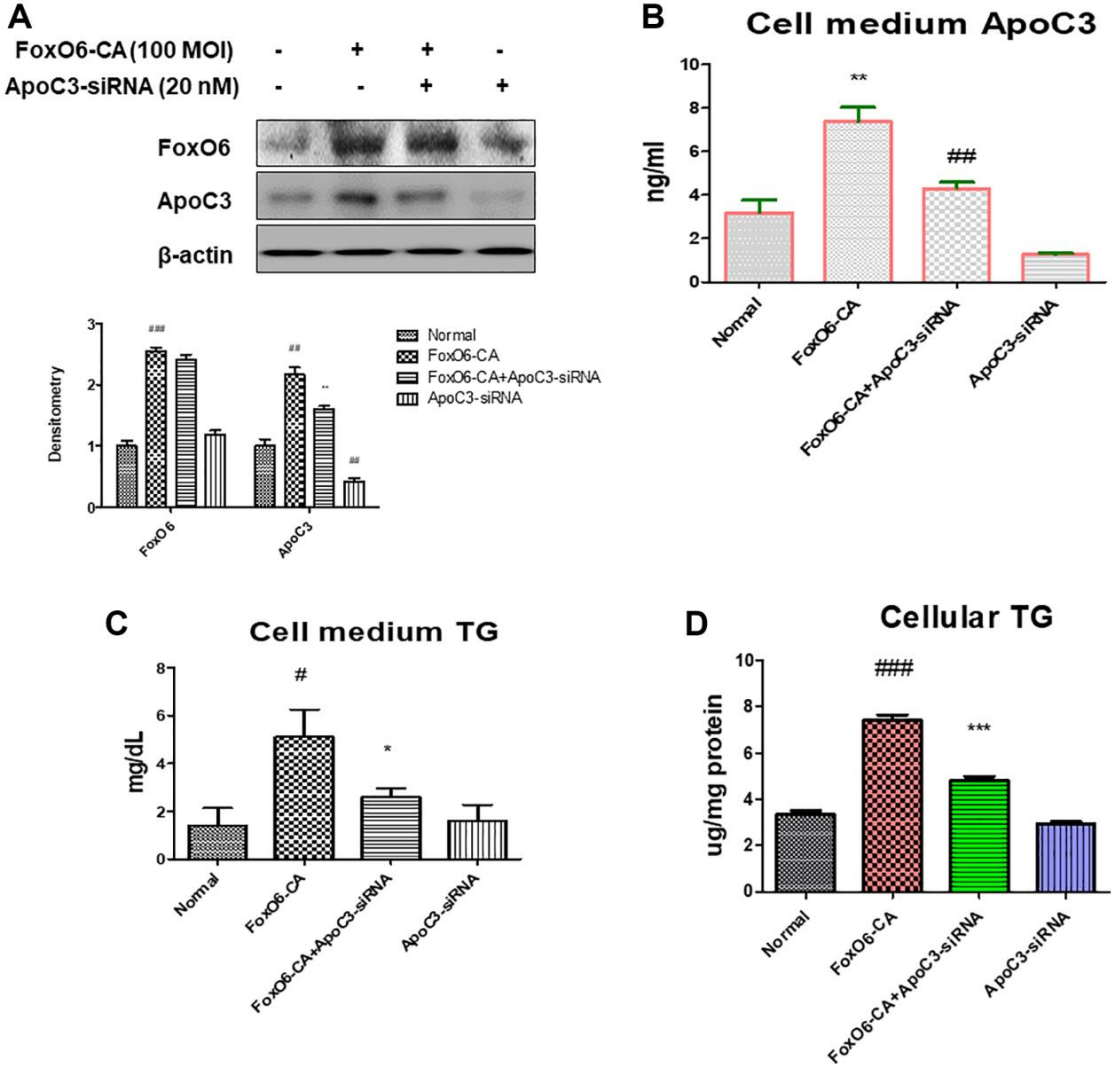
**Deficiency of ApoC3 suppressed FoxO6-mediated lipid accumulation in AC2F liver cells.** (**A**) AC2F cells were transiently transfected with ApoC3-siRNA (20 nM) for 24 h with or without FoxO6 (100 MOI). Cells were analyzed using western blotting using antibodies against FoxO6, ApoC3, and β-actin. Bars in the densitometry data represent the mean ± S.E., and significance was determined using one-factor ANOVA: ^#^*p* < 0.05, ^##^*p* < 0.01, ^###^*p* < 0.001 vs. Normal; ^**^p < 0.01 vs. FoxO6-CA. (**B**) Levels of ApoC3 were determined in the media of the cells. Three independent experiments were performed and similar results were obtained. Results of one-factor ANOVA: ^**^p < 0.01 vs. non-treated cells;^ ##^*p* < 0.01 vs. FoxO6 virus-treated cells. (**C**) TG level of the media from FoxO6 with ApoC3-siRNA-treated cells. (**D**) Cellular TG concentration, after transfected cells were pre-incubated with ApoC3-siRNA (20 nM) for 24 h with or without FoxO6 (100 MOI), was measured using a colorimetric assay. Results of one-factor ANOVA: ^#^*p* < 0.05, ^###^*p* < 0.001 vs. non-treated cells;^ *^p < 0.05, ^***^p < 0.001 vs. FoxO6 virus-treated cells.

In our investigation, we assessed ApoC3 expression levels in AC2F liver cells following transfection with ApoC3-siRNA. Successful knockdown of ApoC3 was achieved 24 hours after treatment with ApoC3-siRNA at a concentration of 20 nM. Additionally, to further delve into the pivotal role of FoxO6 in lipid accumulation, we employed a FoxO6 siRNA-mediated gene manipulation in AC2F cells. We observed a reduction in the elevated levels of FoxO6-mediated ApoC3 expression upon transfection with ApoC3-siRNA (Figure 5A). Furthermore, a significant increase in ApoC3 levels was detected in the medium of cells transfected with FoxO6-CA. Subsequently, we measured ApoC3 levels in the medium obtained from FoxO6-CA-treated cells following transfection with ApoC3-siRNA (Figure 5B). We then examined FoxO6’s capability to stimulate lipid accumulation in AC2F cells. FoxO6 demonstrated an increase in both the TG level in the media and the cellular TG concentration. However, this FoxO6-induced elevation in TG was attenuated following ApoC3-siRNA treatment (Figure 5C, 5D).

### Regulation of the hepatic lipid accumulation in FoxO6-KO and FoxO6-Tg mice

**Figure 6:**
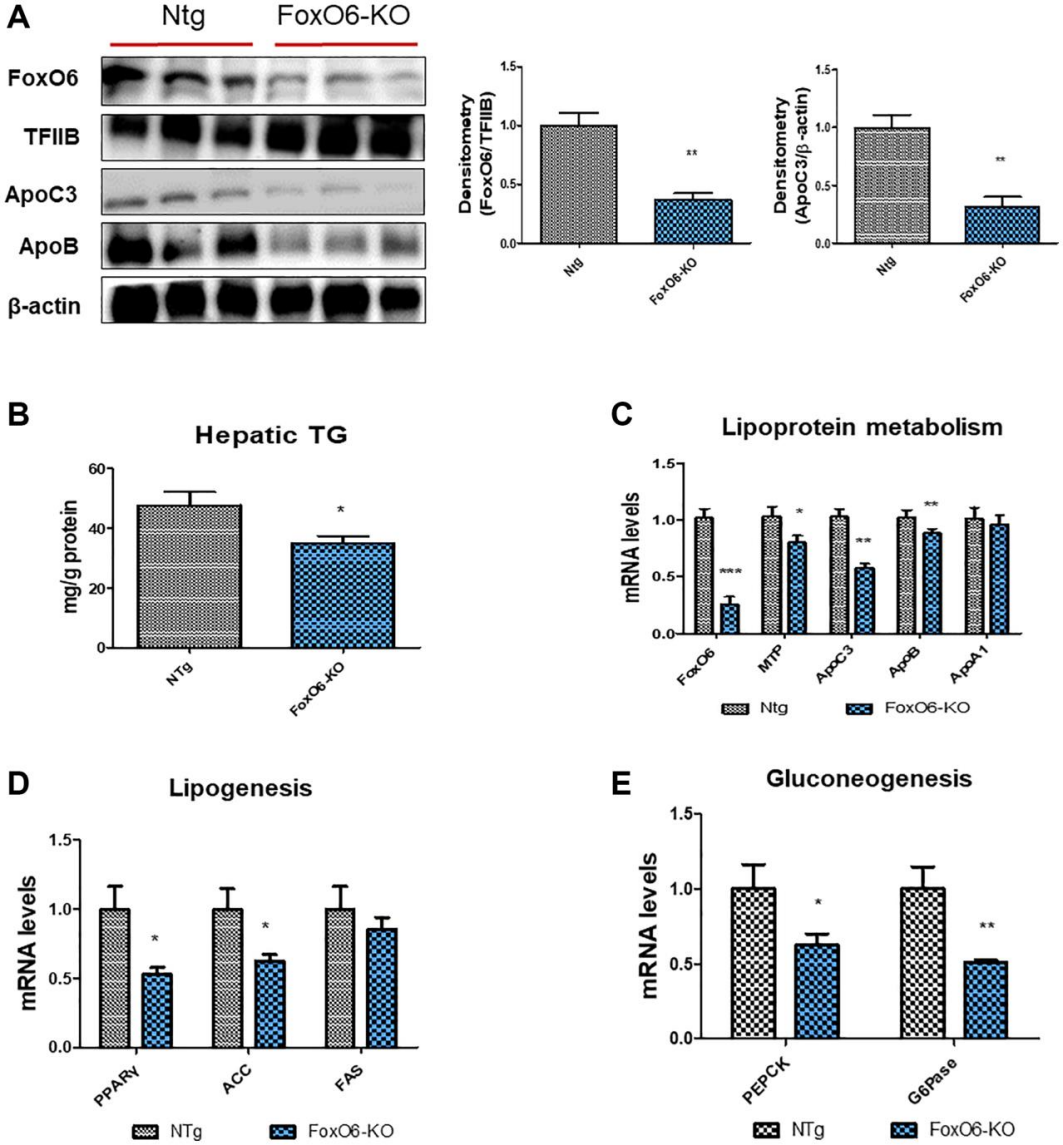
**FoxO6 regulates hepatic lipid accumulation in FoxO6-KO mice.** Mice were fed a high-fat diet at 4 weeks of age for 12 weeks (*n* = 6). (**A**) Western blotting was performed to examine the protein levels of FoxO6, ApoC3, and ApoB in the liver of FoxO6-KO mice. TFIIB was the loading control of the nuclear fractions, whereas β-actin was the loading control of the cytosolic fractions. Results are representative of three independent experiments. Bars in densitometry data represent the mean ± S.E, and significance was determined using one-factor ANOVA: ^**^p < 0.01 vs. NTg. (**B**) Hepatic TG levels in FoxO6-KO mice. One representative result of three experiments yielding similar outcomes for each protein is shown. Results of one-factor ANOVA: ^*^p < 0.05 vs. WT littermates. (**C**) Real-time PCR analyses were performed to measure the mRNA levels of FoxO6, MTP, ApoC3, ApoB, and ApoA1. Three independent experiments were performed and similar results were obtained. Results of one-factor ANOVA: ^*^p < 0.05, ^**^p < 0.01, ^***^p < 0.001 vs. WT littermates. (**D**) Real-time PCR analyses were performed to measure the mRNA levels of PPARγ, ACC, and FAS. Results of one-factor ANOVA: ^*^p < 0.05 vs. WT littermates. (**E**) Real-time PCR analyses were performed for measuring the mRNA levels of PEPCK, and G6Pase. Result of one-factor ANOVA: ^**^p < 0.01 vs. WT littermates.

In a study by Calabuig-Navarro et al. [50], homozygous knockout mice lacking FoxO6 (FoxO6-KO) were generated to elucidate the role of FoxO6 in glucose metabolism. To ascertain the impact of lipid accumulation subsequent to FoxO6 knockout, levels of apolipoproteins and lipogenesis-related genes were evaluated. Our findings revealed a notable reduction in FoxO6 expression within the livers of FoxO6-KO mice (Figure 6A). Additionally, in assessing the effect of ApoC3 and ApoB on FoxO6-KO liver cells, we observed a significant decrease in the expression levels of ApoC3 and ApoB following FoxO6 depletion (Figure 6A). Moreover, hepatic TG contents exhibited a decrease in FoxO6-KO livers when compared to their WT littermates (Figure 6B).

Subsequently, we proceeded to investigate the impact on lipoprotein metabolism using real-time PCR analysis. In FoxO6-KO mice, as anticipated, levels of MTP, ApoC3, and ApoB were notably suppressed (Figure 6C). Additionally, mRNA levels of genes associated with lipogenesis, namely PPARγ, ACC, and FAS, exhibited a decrease in the livers of FoxO6-KO mice (Figure 6D). Furthermore, genes involved in gluconeogenesis, specifically PEPCK and G6Pase, demonstrated reduced expression in the FoxO6-KO livers (Figure 6E). Conversely, in FoxO6-Tg mice, an intensified stimulatory effect on the expression of ApoC3 and ApoB was observed (Supplementary Figure 3A). However, despite this effect, hepatic TG levels were increased in FoxO6-Tg livers compared to their WT littermates (Supplementary Figure 3B).

Subsequent examination of the lipoprotein metabolism via real-time PCR in FoxO6-Tg mice revealed heightened levels of MTP, ApoC3, and ApoB (Supplementary Figure 3C). Additionally, expression levels of lipogenesis genes (PPARγ and ACC) were increased in FoxO6-Tg mice (Supplementary Figure 3D). Notably, genes associated with gluconeogenesis, such as PEPCK and G6Pase, exhibited a significant increase in FoxO6-Tg mice (Supplementary Figure 3E).

## DISCUSSION

Aging constitutes a multifaceted process associated with a spectrum of diseases and metabolic syndromes [51]. Presently, the prevalence of diabetes and obesity is on the rise due to the aging demographic [52]. The onset of NAFLD arises from the excessive accumulation of lipids in the liver, predominantly attributed to escalated caloric intake, particularly from an HFD [53]. Moreover, NAFLD is intricately linked to various age-related metabolic disorders [54]. Previous research has illuminated that during the aging process, there is an upregulation in the expression of genes involved in lipogenesis, a phenomenon attributable to heightened expression levels of FoxO1 and PPARγ. Both FoxO1 and PPARγ are recognized contributors to lipid accumulation [55]. In the current study, it was observed that serum TG and insulin levels exhibited significant elevation in aged rats compared to their younger counterparts, with a further marked increase observed in HFD-fed aged rats (Figure 1). Additionally, histological staining and hepatic triglyceride content were notably augmented in aged rats, further accentuated in HFD-fed aged rats (Figure 1).

FoxO proteins are downstream effectors of the insulin signaling pathway and have been proposed to influence longevity by bolstering resistance against oxidative stress, potentially mitigating oxidative damage and contributing to decelerated aging [56, 57]. Moreover, FoxO proteins are known to mitigate toxicity arising from the aggregation of mutant proteins, and their role in maintaining homeostasis during aging holds direct implications for neurodegenerative diseases [58–60]. We have delineated the molecular mechanisms underlying HG impact on ApoC3 production. Both FoxO6 and ApoC3 protein expression were induced in response to an HFD feeding (Figure 2). However, it was observed that hyperglycemia induced lipid accumulation through the activation of FoxO6 (Figure 3). Our findings suggested that FoxO6 activation elevated the expression of ACC and FAS in liver cells (Figure 4D). Correspondingly, the accumulation of TG increased under these conditions, indicating a regulatory role for FoxO6 in enhancing liver lipid accumulation through the upregulation of lipogenesis gene expression (Figure 4B). PPARγ, known to promote hepatic lipid accumulation, plays a crucial role in hepatic steatosis by upregulating the expression of lipogenesis genes in diet-induced and genetically engineered obese mice [61–64]. Consistently, the knockout of PPARγ in db/db mice noticeably mitigated hepatic steatosis by downregulating the expression of genes such as FAS, SCD1, and ACC [63]. Previous studies have also linked PPARγ to fatty liver development in HFD-fed mice [62]. Our data indicated the binding of FoxO6 to a nucleotide sequence within the ApoC3 promoter (Figure 4C), encompassing insulin-responsive elements (IREs) implicated in mediating the inhibitory effect of insulin on ApoC3 expression [65].

In obese mice, there was an observed increase in FoxO1 expression along with its nuclear localization. This elevation in FoxO1 expression correlated with heightened ApoC3 expression in the liver, concurrent with elevated plasma TG levels and impaired glucose regulation in these mice [16]. Parallel to the effect of FoxO6 on ApoC3 expression in cellular models, activated FoxO6 was demonstrated to elevate hepatic ApoC3 expression, perturbing plasma TG metabolism in liver cells (Figure 4). Moreover, the introduction of a constitutively active form of FoxO6 via adenovirus was associated with augmented cellular ApoC3 production and disrupted TG metabolism in these cells (Figure 4). Additionally, while ApoC3 is primarily produced at low levels in the intestine [66], our study indicated that FoxO6 stimulated hepatic ApoC3 expression in cultured liver cells (Figure 4A). Notably, the capacity of FoxO6 to bind to the ApoC3 promoter was hindered by insulin in liver cells (Figure 4C). Hence, the expression of ApoC3 in the liver is regulated by a mechanism involving HG-dependent FoxO6 action.

ApoC3 functions as a lipid-binding protein primarily found in TG-rich lipoproteins, and its dysregulation has been closely associated with abnormal TG metabolism [16, 43, 67–71]. While ApoC3’s pivotal role in the pathogenesis of hypertriglyceridemia is well-established, its precise biological function in NAFLD remains elusive. Genetic mutations disrupting the carboxyl lipid-binding domain of ApoC3 lead to loss-of-function mutations. These mutations hinder ApoC3’s ability to stimulate the formation of TG-enriched luminal lipid droplets (LLD) and the secretion of VLDL1 [25, 72, 73]. Additionally, a study by Ginsberg and Fisher [74] reported that in comparing ApoC3-Tg mice to WT mice, the levels of ApoB mRNA did not exhibit significant alterations.

Insulin resistance has been implicated in the reduction of excessive TG production within the liver, attenuating steatosis. This attenuation coincides with the elevation of ApoC3 and ApoB expression observed in FoxO6-Tg mice (Supplementary Figure 3). Moreover, FoxO6 has been associated with heightened lipogenesis, contributing to increased fat accumulation specifically within the liver of FoxO6-Tg mice (Supplementary Figure 3). Detailed investigations into the underlying molecular mechanisms revealed a significant increase in the expression of lipogenesis genes within the livers of FoxO6-Tg mice (Supplementary Figure 3). Depletion of FoxO6 significantly diminished the levels of ApoC3 and ApoB (Figure 6A), and hepatic TG content was notably reduced in the livers of FoxO6-KO mice (Figure 6B). Fatty liver disease is closely associated with obesity [75] and insulin resistance. The link between hepatic steatosis and insulin resistance, observed both in humans [76] and animal models [77, 78], suggests that insulin resistance might play a crucial role in the pathogenesis of obesity-induced fatty liver disease. Our current research further demonstrates that ApoC3 can modulate hepatic fat accumulation by regulating FoxO6 activity.

Dysfunctional gluconeogenesis serves as a significant indicator of liver insulin resistance. Our investigation highlighted a substantial increase in the mRNA levels of key gluconeogenesis markers, PEPCK and G6Pase, within the liver of aged mice displaying liver insulin resistance (Figure 2 and Supplementary Figure 2). In our study, we uncovered the involvement of gluconeogenesis and FoxO6 in the context of insulin resistance. Moreover, treatment with HG notably elevated FoxO6 protein expression levels along with a significant increase in G6Pase and PEPCK mRNA levels. Intriguingly, the FoxO6-KO attenuated the upregulation of these genes associated with gluconeogenesis (Figure 6E). Based on these findings, we proposed the hypothesis that hyperglycemia-mediated FoxO6 plays a pivotal role in hindering insulin signaling, subsequently prompting the induction of gluconeogenesis-related genes in conditions marked by insulin resistance.

We have identified the FoxO6 and ApoC3 signaling pathway as a key hepatic target involved in the modulation of glucose-induced enhancement of TG levels (Figure 3). The suppression of FoxO6 expression and its downstream target gene ApoC3 in the liver results in reduced ApoC3 concentrations in the plasma, underscoring the therapeutic potential of targeting FoxO6 in glucose-related effects (Figure 5).

To summarize, the activation of FoxO6 via inhibition of the IRS/Akt pathway induces gluconeogenesis in aged rats fed an HFD, subsequently leading to upregulated hepatic ApoC3 expression. This elevation of ApoC3 levels contributes to hyperlipidemia and hepatic steatosis, potentially exacerbating the aging process. Under hyperglycemic conditions, both *in vivo* and *in vitro*, FoxO6 demonstrates an increased capacity to augment ApoC3 expression (Figure 4E).

Furthermore, in the context of hyperglycemia, hepatic expression and activation of FoxO6 significantly contribute to increased ApoC3 production, impairing plasma TG metabolism associated with aging in HFD-fed conditions. Conversely, targeted inhibition of FoxO6 holds promise in ameliorating age-related dyslipidemia by restraining ApoC3 production in the liver, thereby suppressing its transcriptional activity. In conclusion, the upregulation of ApoC3 via FoxO6 activation leads to the induction of hyperlipidemia and hepatic steatosis in aged rats subjected to an HFD. This discovery unveils a potential novel molecular target for therapeutic strategies against hepatic steatosis during the aging process, offering insights into its molecular and cellular underpinnings, particularly its association with FoxO6-mediated ApoC3 upregulation.

## MATERIALS AND METHODS

### Reagents

Chemical reagents were obtained from Sigma-Aldrich (St. Louis, MO, USA). All primary (diluted to 1:1,000) and secondary (diluted to 1:10,000) antibodies were obtained from Santa Cruz Biotechnology (Santa Cruz, CA, USA). FoxO6 and phosphorylated (Ser184) of FoxO6 antibodies were obtained from Dr. Dong (University of Pittsburgh, Pittsburgh, PA, USA).

### Animal studies

Sprague Dawley young male rats (6-month-old) and elderly rats (22-month-old) were purchased from Samtako (Gueonggi-do, Korea) and acclimated to the animal care facility for 7 days before the experiments. Animals were housed in an air-conditioned atmosphere under a 12-h light/dark cycle and were provided free access to standard rodent chow (Samtako) and water. To induce obesity, rats were fed a high-fat diet (60% fat, Research Diets Inc.; D12492) for 14 days. All procedures were approved by Pusan National University and performed by animal committee (PNU-2017-1534).

The livers from FoxO6-Knockout (KO) and FoxO6-Transgenic (Tg) mice were obtained from Dr. H. Henry Dong (University of Pittsburgh Medical Center, Pittsburgh, PA, USA).

### Cell culture

AC2F (rat hepatocellular carcinoma) cells were obtained from the American Type Culture Collection (Rockville, MD, USA), as described previously [55].

### Serum biochemical analyses

Serum samples were prepared by centrifugation (4°C, 2000 × g for 15 min) after euthanasia. Glucose, free fatty acid (FFA), HDL, LDL, and TG level were measured using serum kits from Bioassay Systems (Hayward, CA, USA). The insulin levels used to rat ELISA kits (SHIBAYAGI, Shibukawa, Japan) and rat ApoC3 levels (Novus Biologicals, CO, USA). HOMA-IR was calculated using the HOMA2 calculator [79].

### Measurement of liver TG

Liver tissues and cells were homogenized in phosphate-buffered saline (PBS), as described previously [55].

### Histological analysis

Liver tissue was fixed in paraffin-embedded sections were stained with hematoxylin and eosin (H&E), following the method outlined in an earlier reference [55].

### Immunohistochemistry analysis

For immunostaining, liver sections were treated with 0.6% H_2_O_2_ in Tris-buffered saline (TBS; pH 7.5) to block endogenous peroxidase for 15 min at room temperature, as described previously [55].

### Immunofluorescence analysis

AC2F cells were seeded at a density of 1 × 10^4^ cells per well in a 12-well plate, following the method outlined in an earlier reference [80].

### Protein extraction

All solutions, tubes, and centrifuges were maintained at 0–4°C. A total of 1 g of liver was homogenized with hypotonic lysis buffer, as described previously [81].

### Western blot analysis

Western blotting was performed in cytosolic and nuclear fraction, as described previously [55].

### Isolation and quantitative real-time PCR

Tissue RNA was purified using RiboEx Total RNA (GeneAll, Republic of Korea), following the method outlined in an earlier reference [55].

### Chromatin immunoprecipitation (ChIP) assay

ChIP was used to study the interaction between FoxO6 and ApoC3 promoter DNA in the cells, as described previously [81]. ApoC3 promoter-specific primers (forward: 5′-ctctcacagccaggacagtt-3′ and reverse: 5′-agctgccagaagagttgaga-3′), which flank the consensus FoxO6-binding sites in rat ApoC3 promoters.

### Transfection of small interfering RNA (siRNA)

Transfection was performed using Lipofectamine 2000 reagent (Invitrogen). The liver cells were treated with scrambled-siRNA or ApoC3-siRNA-Lipofectamine complexes (20 nM) obtained from a commercial source (IDT), following the method outlined in an earlier reference [81].

### Statistical analysis

Analysis of variance (ANOVA) was used to analyze differences among the three or more groups, as described previously [81].

## Supplementary Materials

Supplementary Figures

## References

[1] Guan Y, Zhang Y, Breyer MD. The Role of PPARs in the Transcriptional Control of Cellular Processes. Drug News Perspect. 2002; 15:147–54. 10.1358/dnp.2002.15.3.840011 12677257

[2] Lin HV, Accili D. Hormonal regulation of hepatic glucose production in health and disease. Cell Metab. 2011; 14:9–19. 10.1016/j.cmet.2011.06.003 21723500 PMC3131084

[3] Ferré P. The biology of peroxisome proliferator-activated receptors: relationship with lipid metabolism and insulin sensitivity. Diabetes. 2004 (Suppl 1); 53:S43–50. 10.2337/diabetes.53.2007.s43 14749265

[4] Wree A, Broderick L, Canbay A, Hoffman HM, Feldstein AE. From NAFLD to NASH to cirrhosis-new insights into disease mechanisms. Nat Rev Gastroenterol Hepatol. 2013; 10:627–36. 10.1038/nrgastro.2013.149 23958599

[5] Van Der Heide LP, Hoekman MF, Smidt MP. The ins and outs of FoxO shuttling: mechanisms of FoxO translocation and transcriptional regulation. Biochem J. 2004; 380:297–309. 10.1042/BJ20040167 15005655 PMC1224192

[6] Accili D, Arden KC. FoxOs at the crossroads of cellular metabolism, differentiation, and transformation. Cell. 2004; 117:421–6. 10.1016/s0092-8674(04)00452-0 15137936

[7] Barthel A, Schmoll D, Unterman TG. FoxO proteins in insulin action and metabolism. Trends Endocrinol Metab. 2005; 16:183–9. 10.1016/j.tem.2005.03.010 15860415

[8] Biggs WH 3rd, Meisenhelder J, Hunter T, Cavenee WK, Arden KC. Protein kinase B/Akt-mediated phosphorylation promotes nuclear exclusion of the winged helix transcription factor FKHR1. Proc Natl Acad Sci U S A. 1999; 96:7421–6. 10.1073/pnas.96.13.7421 10377430 PMC22101

[9] Kawamori D, Kaneto H, Nakatani Y, Matsuoka TA, Matsuhisa M, Hori M, Yamasaki Y. The forkhead transcription factor Foxo1 bridges the JNK pathway and the transcription factor PDX-1 through its intracellular translocation. J Biol Chem. 2006; 281:1091–8. 10.1074/jbc.M508510200 16282329

[10] Martinez SC, Tanabe K, Cras-Méneur C, Abumrad NA, Bernal-Mizrachi E, Permutt MA. Inhibition of Foxo1 protects pancreatic islet beta-cells against fatty acid and endoplasmic reticulum stress-induced apoptosis. Diabetes. 2008; 57:846–59. 10.2337/db07-0595 18174526

[11] Bose SK, Kim H, Meyer K, Wolins N, Davidson NO, Ray R. Forkhead box transcription factor regulation and lipid accumulation by hepatitis C virus. J Virol. 2014; 88:4195–203. 10.1128/JVI.03327-13 24478438 PMC3993747

[12] Kamagate A, Qu S, Perdomo G, Su D, Kim DH, Slusher S, Meseck M, Dong HH. FoxO1 mediates insulin-dependent regulation of hepatic VLDL production in mice. J Clin Invest. 2008; 118:2347–64. 10.1172/JCI32914 18497885 PMC2391277

[13] Polvani S, Tarocchi M, Galli A. PPARγ and Oxidative Stress: Con(β) Catenating NRF2 and FOXO. PPAR Res. 2012; 2012:641087. 10.1155/2012/641087 22481913 PMC3317010

[14] Dowell P, Otto TC, Adi S, Lane MD. Convergence of peroxisome proliferator-activated receptor gamma and Foxo1 signaling pathways. J Biol Chem. 2003; 278:45485–91. 10.1074/jbc.M309069200 12966085

[15] Kim DH, Zhang T, Lee S, Calabuig-Navarro V, Yamauchi J, Piccirillo A, Fan Y, Uppala R, Goetzman E, Dong HH. FoxO6 integrates insulin signaling with MTP for regulating VLDL production in the liver. Endocrinology. 2014; 155:1255–67. 10.1210/en.2013-1856 24437489 PMC3959596

[16] Altomonte J, Cong L, Harbaran S, Richter A, Xu J, Meseck M, Dong HH. Foxo1 mediates insulin action on apoC-III and triglyceride metabolism. J Clin Invest. 2004; 114:1493–503. 10.1172/JCI19992 15546000 PMC525736

[17] Lai CQ, Parnell LD, Ordovas JM. The APOA1/C3/A4/A5 gene cluster, lipid metabolism and cardiovascular disease risk. Curr Opin Lipidol. 2005; 16:153–66. 10.1097/01.mol.0000162320.54795.68 15767855

[18] van Dijk KW, Rensen PC, Voshol PJ, Havekes LM. The role and mode of action of apolipoproteins CIII and AV: synergistic actors in triglyceride metabolism? Curr Opin Lipidol. 2004; 15:239–46. 10.1097/00041433-200406000-00002 15166778

[19] Jong MC, Hofker MH, Havekes LM. Role of ApoCs in lipoprotein metabolism: functional differences between ApoC1, ApoC2, and ApoC3. Arterioscler Thromb Vasc Biol. 1999; 19:472–84. 10.1161/01.atv.19.3.472 10073946

[20] Wang CS, McConathy WJ, Kloer HU, Alaupovic P. Modulation of lipoprotein lipase activity by apolipoproteins. Effect of apolipoprotein C-III. J Clin Invest. 1985; 75:384–90. 10.1172/JCI111711 3973011 PMC423500

[21] McConathy WJ, Gesquiere JC, Bass H, Tartar A, Fruchart JC, Wang CS. Inhibition of lipoprotein lipase activity by synthetic peptides of apolipoprotein C-III. J Lipid Res. 1992; 33:995–1003. 1431591

[22] Kinnunen PK, Ehnolm C. Effect of serum and C-apoproteins from very low density lipoproteins on human postheparin plasma hepatic lipase. FEBS Lett. 1976; 65:354–7. 10.1016/0014-5793(76)80145-7 182536

[23] Quarfordt SH, Michalopoulos G, Schirmer B. The effect of human C apolipoproteins on the in vitro hepatic metabolism of triglyceride emulsions in the rat. J Biol Chem. 1982; 257:14642–7. 7174660

[24] Mann CJ, Troussard AA, Yen FT, Hannouche N, Najib J, Fruchart JC, Lotteau V, André P, Bihain BE. Inhibitory effects of specific apolipoprotein C-III isoforms on the binding of triglyceride-rich lipoproteins to the lipolysis-stimulated receptor. J Biol Chem. 1997; 272:31348–54. 10.1074/jbc.272.50.31348 9395464

[25] Gordts PL, Nock R, Son NH, Ramms B, Lew I, Gonzales JC, Thacker BE, Basu D, Lee RG, Mullick AE, Graham MJ, Goldberg IJ, Crooke RM, et al. ApoC-III inhibits clearance of triglyceride-rich lipoproteins through LDL family receptors. J Clin Invest. 2016; 126:2855–66. 10.1172/JCI86610 27400128 PMC4966320

[26] Qin W, Sundaram M, Wang Y, Zhou H, Zhong S, Chang CC, Manhas S, Yao EF, Parks RJ, McFie PJ, Stone SJ, Jiang ZG, Wang C, et al. Missense mutation in APOC3 within the C-terminal lipid binding domain of human ApoC-III results in impaired assembly and secretion of triacylglycerol-rich very low density lipoproteins: evidence that ApoC-III plays a major role in the formation of lipid precursors within the microsomal lumen. J Biol Chem. 2011; 286:27769–80. 10.1074/jbc.M110.203679 21676879 PMC3149367

[27] Chan DC, Watts GF, Nguyen MN, Barrett PH. Apolipoproteins C-III and A-V as predictors of very-low-density lipoprotein triglyceride and apolipoprotein B-100 kinetics. Arterioscler Thromb Vasc Biol. 2006; 26:590–6. 10.1161/01.ATV.0000203519.25116.54 16410456

[28] Taskinen MR, Adiels M, Westerbacka J, Söderlund S, Kahri J, Lundbom N, Lundbom J, Hakkarainen A, Olofsson SO, Orho-Melander M, Borén J. Dual metabolic defects are required to produce hypertriglyceridemia in obese subjects. Arterioscler Thromb Vasc Biol. 2011; 31:2144–50. 10.1161/ATVBAHA.111.224808 21778423

[29] Cohn JS, Patterson BW, Uffelman KD, Davignon J, Steiner G. Rate of production of plasma and very-low-density lipoprotein (VLDL) apolipoprotein C-III is strongly related to the concentration and level of production of VLDL triglyceride in male subjects with different body weights and levels of insulin sensitivity. J Clin Endocrinol Metab. 2004; 89:3949–55. 10.1210/jc.2003-032056 15292332

[30] Ito Y, Azrolan N, O'Connell A, Walsh A, Breslow JL. Hypertriglyceridemia as a result of human apo CIII gene expression in transgenic mice. Science. 1990; 249:790–3. 10.1126/science.2167514 2167514

[31] Maeda N, Li H, Lee D, Oliver P, Quarfordt SH, Osada J. Targeted disruption of the apolipoprotein C-III gene in mice results in hypotriglyceridemia and protection from postprandial hypertriglyceridemia. J Biol Chem. 1994; 269:23610–6. 8089130

[32] Gerritsen G, Rensen PC, Kypreos KE, Zannis VI, Havekes LM, Willems van Dijk K. ApoC-III deficiency prevents hyperlipidemia induced by apoE overexpression. J Lipid Res. 2005; 46:1466–73. 10.1194/jlr.M400479-JLR200 15863838

[33] Jong MC, Rensen PC, Dahlmans VE, van der Boom H, van Berkel TJ, Havekes LM. Apolipoprotein C-III deficiency accelerates triglyceride hydrolysis by lipoprotein lipase in wild-type and apoE knockout mice. J Lipid Res. 2001; 42:1578–85. 11590213

[34] Pollin TI, Damcott CM, Shen H, Ott SH, Shelton J, Horenstein RB, Post W, McLenithan JC, Bielak LF, Peyser PA, Mitchell BD, Miller M, O'Connell JR, Shuldiner AR. A null mutation in human APOC3 confers a favorable plasma lipid profile and apparent cardioprotection. Science. 2008; 322:1702–5. 10.1126/science.1161524 19074352 PMC2673993

[35] Jørgensen AB, Frikke-Schmidt R, Nordestgaard BG, Tybjærg-Hansen A. Loss-of-function mutations in APOC3 and risk of ischemic vascular disease. N Engl J Med. 2014; 371:32–41. 10.1056/NEJMoa1308027 24941082

[36] Graham MJ, Lee RG, Bell TA 3rd, Fu W, Mullick AE, Alexander VJ, Singleton W, Viney N, Geary R, Su J, Baker BF, Burkey J, Crooke ST, Crooke RM. Antisense oligonucleotide inhibition of apolipoprotein C-III reduces plasma triglycerides in rodents, nonhuman primates, and humans. Circ Res. 2013; 112:1479–90. 10.1161/CIRCRESAHA.111.300367 23542898

[37] Gaudet D, Brisson D, Tremblay K, Alexander VJ, Singleton W, Hughes SG, Geary RS, Baker BF, Graham MJ, Crooke RM, Witztum JL. Targeting APOC3 in the familial chylomicronemia syndrome. N Engl J Med. 2014; 371:2200–6. 10.1056/NEJMoa1400284 25470695

[38] Norata GD, Tsimikas S, Pirillo A, Catapano AL. Apolipoprotein C-III: From Pathophysiology to Pharmacology. Trends Pharmacol Sci. 2015; 36:675–87. 10.1016/j.tips.2015.07.001 26435212

[39] Khetarpal SA, Qamar A, Millar JS, Rader DJ. Targeting ApoC-III to Reduce Coronary Disease Risk. Curr Atheroscler Rep. 2016; 18:54. 10.1007/s11883-016-0609-y 27443326

[40] Kawakami A, Aikawa M, Alcaide P, Luscinskas FW, Libby P, Sacks FM. Apolipoprotein CIII induces expression of vascular cell adhesion molecule-1 in vascular endothelial cells and increases adhesion of monocytic cells. Circulation. 2006; 114:681–7. 10.1161/CIRCULATIONAHA.106.622514 16894036

[41] Abe Y, Kawakami A, Osaka M, Uematsu S, Akira S, Shimokado K, Sacks FM, Yoshida M. Apolipoprotein CIII induces monocyte chemoattractant protein-1 and interleukin 6 expression via Toll-like receptor 2 pathway in mouse adipocytes. Arterioscler Thromb Vasc Biol. 2010; 30:2242–8. 10.1161/ATVBAHA.110.210427 20829510 PMC3203842

[42] Pollex RL, Ban MR, Young TK, Bjerregaard P, Anand SS, Yusuf S, Zinman B, Harris SB, Hanley AJ, Connelly PW, Huff MW, Hegele RA. Association between the -455T>C promoter polymorphism of the APOC3 gene and the metabolic syndrome in a multi-ethnic sample. BMC Med Genet. 2007; 8:80. 10.1186/1471-2350-8-80 18096054 PMC2241585

[43] Richart C, Auguet T, Terra X. Apolipoprotein C3 gene variants in nonalcoholic fatty liver disease. N Engl J Med. 2010; 363:193–4. 10.1056/NEJMc1005265 20647217

[44] Waterworth DM, Ribalta J, Nicaud V, Dallongeville J, Humphries SE, Talmud P. ApoCIII gene variants modulate postprandial response to both glucose and fat tolerance tests. Circulation. 1999; 99:1872–7. 10.1161/01.cir.99.14.1872 10199885

[45] Kaur J. A comprehensive review on metabolic syndrome. Cardiol Res Pract. 2014; 2014:943162. 10.1155/2014/943162 24711954 PMC3966331

[46] Meyer C, Nadkarni V, Stumvoll M, Gerich J. Human kidney free fatty acid and glucose uptake: evidence for a renal glucose-fatty acid cycle. Am J Physiol. 1997; 273:E650–4. 10.1152/ajpendo.1997.273.3.E650 9316458

[47] Kang HM, Ahn SH, Choi P, Ko YA, Han SH, Chinga F, Park AS, Tao J, Sharma K, Pullman J, Bottinger EP, Goldberg IJ, Susztak K. Defective fatty acid oxidation in renal tubular epithelial cells has a key role in kidney fibrosis development. Nat Med. 2015; 21:37–46. 10.1038/nm.3762 25419705 PMC4444078

[48] Ha S, Kim MJ, Kim DH, Kim BM, Chung KW, Chung HY. Short-term intake of high fat diet aggravates renal fibrosis in aged Sprague-Dawley rats. Exp Gerontol. 2020; 142:111108. 10.1016/j.exger.2020.111108 33130113

[49] Kim DH, Perdomo G, Zhang T, Slusher S, Lee S, Phillips BE, Fan Y, Giannoukakis N, Gramignoli R, Strom S, Ringquist S, Dong HH. FoxO6 integrates insulin signaling with gluconeogenesis in the liver. Diabetes. 2011; 60:2763–74. 10.2337/db11-0548 21940782 PMC3198083

[50] Calabuig-Navarro V, Yamauchi J, Lee S, Zhang T, Liu YZ, Sadlek K, Coudriet GM, Piganelli JD, Jiang CL, Miller R, Lowe M, Harashima H, Dong HH. Forkhead Box O6 (FoxO6) Depletion Attenuates Hepatic Gluconeogenesis and Protects against Fat-induced Glucose Disorder in Mice. J Biol Chem. 2015; 290:15581–94. 10.1074/jbc.M115.650994 25944898 PMC4505471

[51] Chung HY, Kim DH, Lee EK, Chung KW, Chung S, Lee B, Seo AY, Chung JH, Jung YS, Im E, Lee J, Kim ND, Choi YJ, et al. Redefining Chronic Inflammation in Aging and Age-Related Diseases: Proposal of the Senoinflammation Concept. Aging Dis. 2019; 10:367–82. 10.14336/AD.2018.0324 31011483 PMC6457053

[52] Park MH, Kim DH, Lee EK, Kim ND, Im DS, Lee J, Yu BP, Chung HY. Age-related inflammation and insulin resistance: a review of their intricate interdependency. Arch Pharm Res. 2014; 37:1507–14. 10.1007/s12272-014-0474-6 25239110 PMC4246128

[53] Ip YT, Davis RJ. Signal transduction by the c-Jun N-terminal kinase (JNK)--from inflammation to development. Curr Opin Cell Biol. 1998; 10:205–19. 10.1016/s0955-0674(98)80143-9 9561845

[54] Sheedfar F, Di Biase S, Koonen D, Vinciguerra M. Liver diseases and aging: friends or foes? Aging Cell. 2013; 12:950–4. 10.1111/acel.12128 23815295

[55] Kim DH, Ha S, Choi YJ, Dong HH, Yu BP, Chung HY. Altered FoxO1 and PPARγ interaction in age-related ER stress-induced hepatic steatosis. Aging (Albany NY). 2019; 11:4125–44. 10.18632/aging.102042 31246177 PMC6628996

[56] Balaban RS, Nemoto S, Finkel T. Mitochondria, oxidants, and aging. Cell. 2005; 120:483–95. 10.1016/j.cell.2005.02.001 15734681

[57] Panici JA, Harper JM, Miller RA, Bartke A, Spong A, Masternak MM. Early life growth hormone treatment shortens longevity and decreases cellular stress resistance in long-lived mutant mice. FASEB J. 2010; 24:5073–9. 10.1096/fj.10-163253 20720157 PMC2992365

[58] Cohen E, Bieschke J, Perciavalle RM, Kelly JW, Dillin A. Opposing activities protect against age-onset proteotoxicity. Science. 2006; 313:1604–10. 10.1126/science.1124646 16902091

[59] Hsu AL, Murphy CT, Kenyon C. Regulation of aging and age-related disease by DAF-16 and heat-shock factor. Science. 2003; 300:1142–5. 10.1126/science.1083701 12750521

[60] Morley JF, Brignull HR, Weyers JJ, Morimoto RI. The threshold for polyglutamine-expansion protein aggregation and cellular toxicity is dynamic and influenced by aging in Caenorhabditis elegans. Proc Natl Acad Sci U S A. 2002; 99:10417–22. 10.1073/pnas.152161099 12122205 PMC124929

[61] Ables GP. Update on pparγ and nonalcoholic Fatty liver disease. PPAR Res. 2012; 2012:912351. 10.1155/2012/912351 22966224 PMC3431124

[62] Inoue M, Ohtake T, Motomura W, Takahashi N, Hosoki Y, Miyoshi S, Suzuki Y, Saito H, Kohgo Y, Okumura T. Increased expression of PPARgamma in high fat diet-induced liver steatosis in mice. Biochem Biophys Res Commun. 2005; 336:215–22. 10.1016/j.bbrc.2005.08.070 16125673

[63] Matsusue K, Haluzik M, Lambert G, Yim SH, Gavrilova O, Ward JM, Brewer B Jr, Reitman ML, Gonzalez FJ. Liver-specific disruption of PPARgamma in leptin-deficient mice improves fatty liver but aggravates diabetic phenotypes. J Clin Invest. 2003; 111:737–47. 10.1172/JCI17223 12618528 PMC151902

[64] Yu S, Matsusue K, Kashireddy P, Cao WQ, Yeldandi V, Yeldandi AV, Rao MS, Gonzalez FJ, Reddy JK. Adipocyte-specific gene expression and adipogenic steatosis in the mouse liver due to peroxisome proliferator-activated receptor gamma1 (PPARgamma1) overexpression. J Biol Chem. 2003; 278:498–505. 10.1074/jbc.M210062200 12401792

[65] Li WW, Dammerman MM, Smith JD, Metzger S, Breslow JL, Leff T. Common genetic variation in the promoter of the human apo CIII gene abolishes regulation by insulin and may contribute to hypertriglyceridemia. J Clin Invest. 1995; 96:2601–5. 10.1172/JCI118324 8675624 PMC185964

[66] Haddad IA, Ordovas JM, Fitzpatrick T, Karathanasis SK. Linkage, evolution, and expression of the rat apolipoprotein A-I, C-III, and A-IV genes. J Biol Chem. 1986; 261:13268–77. 3020028

[67] Waterworth DM, Hubacek JA, Pitha J, Kovar J, Poledne R, Humphries SE, Talmud PJ. Plasma levels of remnant particles are determined in part by variation in the APOC3 gene insulin response element and the APOCI-APOE cluster. J Lipid Res. 2000; 41:1103–9. 10884292

[68] Hegele RA, Connelly PW, Hanley AJ, Sun F, Harris SB, Zinman B. Common genomic variation in the APOC3 promoter associated with variation in plasma lipoproteins. Arterioscler Thromb Vasc Biol. 1997; 17:2753–8. 10.1161/01.atv.17.11.2753 9409252

[69] Chen M, Breslow JL, Li W, Leff T. Transcriptional regulation of the apoC-III gene by insulin in diabetic mice: correlation with changes in plasma triglyceride levels. J Lipid Res. 1994; 35:1918–24. 7868970

[70] Ebara T, Ramakrishnan R, Steiner G, Shachter NS. Chylomicronemia due to apolipoprotein CIII overexpression in apolipoprotein E-null mice. Apolipoprotein CIII-induced hypertriglyceridemia is not mediated by effects on apolipoprotein E. J Clin Invest. 1997; 99:2672–81. 10.1172/JCI119456 9169497 PMC508113

[71] Talmud PJ, Humphries SE. Apolipoprotein C-III gene variation and dyslipidaemia. Curr Opin Lipidol. 1997; 8:154–8. 10.1097/00041433-199706000-00005 9211063

[72] Yao Z. Human apolipoprotein C-III - a new intrahepatic protein factor promoting assembly and secretion of very low density lipoproteins. Cardiovasc Hematol Disord Drug Targets. 2012; 12:133–40. 10.2174/1871529x11202020133 23030451

[73] Sundaram M, Zhong S, Bou Khalil M, Zhou H, Jiang ZG, Zhao Y, Iqbal J, Hussain MM, Figeys D, Wang Y, Yao Z. Functional analysis of the missense APOC3 mutation Ala23Thr associated with human hypotriglyceridemia. J Lipid Res. 2010; 51:1524–34. 10.1194/jlr.M005108 20097930 PMC3035516

[74] Ginsberg HN, Fisher EA. The ever-expanding role of degradation in the regulation of apolipoprotein B metabolism. J Lipid Res. 2009; 50:S162–6. 10.1194/jlr.R800090-JLR200 19050312 PMC2674708

[75] Silverman JF, Pories WJ, Caro JF. Liver pathology in diabetes mellitus and morbid obesity. Clinical, pathological, and biochemical considerations. Pathol Annu. 1989; 24:275–302. 2654841

[76] Marceau P, Biron S, Hould FS, Marceau S, Simard S, Thung SN, Kral JG. Liver pathology and the metabolic syndrome X in severe obesity. J Clin Endocrinol Metab. 1999; 84:1513–7. 10.1210/jcem.84.5.5661 10323371

[77] Uysal KT, Wiesbrock SM, Marino MW, Hotamisligil GS. Protection from obesity-induced insulin resistance in mice lacking TNF-alpha function. Nature. 1997; 389:610–4. 10.1038/39335 9335502

[78] Shimomura I, Bashmakov Y, Horton JD. Increased levels of nuclear SREBP-1c associated with fatty livers in two mouse models of diabetes mellitus. J Biol Chem. 1999; 274:30028–32. 10.1074/jbc.274.42.30028 10514488

[79] Wallace TM, Levy JC, Matthews DR. An increase in insulin sensitivity and basal beta-cell function in diabetic subjects treated with pioglitazone in a placebo-controlled randomized study. Diabet Med. 2004; 21:568–76. 10.1111/j.1464-5491.2004.01218.x 15154941

[80] Chung JH, Kim DH, Kim YS, Son BS, Kim D, Hwang C, Shin D, Noh SG, Han JH, Kim DK, Kim JH, Koo JS, Chung HY, Yoon SH. Upregulation of P21-Activated Kinase 1 (PAK1)/CREB Axis in Squamous Non-Small Cell Lung Carcinoma. Cell Physiol Biochem. 2018; 50:304–16. 10.1159/000494007 30282071

[81] Kim DH, Lee B, Lee J, Kim ME, Lee JS, Chung JH, Yu BP, Dong HH, Chung HY. FoxO6-mediated IL-1β induces hepatic insulin resistance and age-related inflammation via the TF/PAR2 pathway in aging and diabetic mice. Redox Biol. 2019; 24:101184. 10.1016/j.redox.2019.101184 30974318 PMC6454229

