# Supplementary material for: FoxO6-mediated ApoC3 upregulation promotes hepatic steatosis and hyperlipidemia in aged rats fed a high-fat diet
Source: Aging (Albany NY). 2024 Mar 3;16(5):4095–115. doi: 10.18632/aging.205610 (PMC10968681; doi:10.18632/aging.205610)
Supplement: Supplementary Figures [file aging-16-205610-s001.pdf]

## SUPPLEMENTARY FIGURES

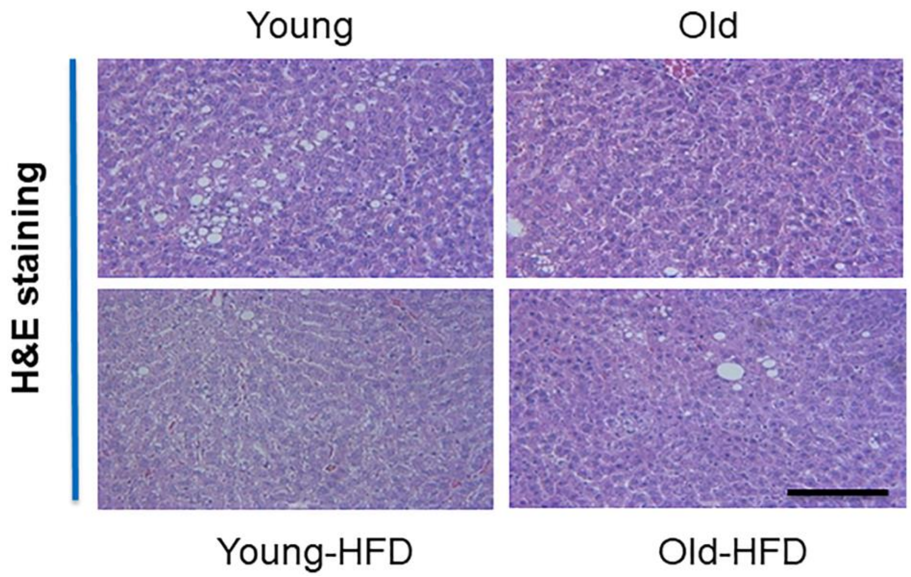


**Supplementary Figure 1. HFD feeding induced liver damage in aged rats.** Representative H&E staining shows increased vacuoles in liver tubules during aging. Scale bar: 100 µm.

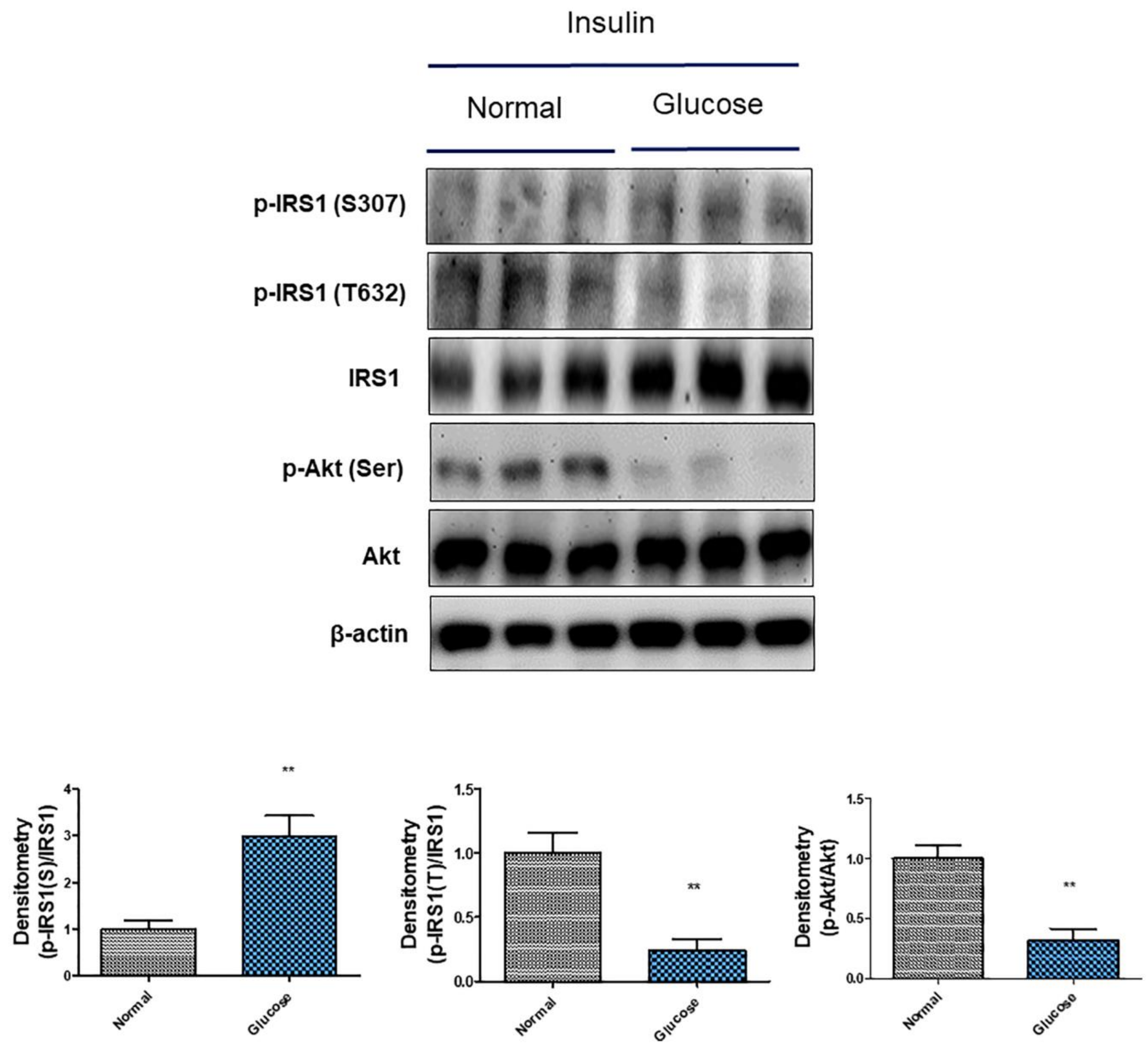


**Supplementary Figure 2. High-glucose suppressed the insulin signaling.** Western blotting was used to detect p-IRS1 (Ser307), p-IRS1 (Tyr632), IRS1, p-Akt (Ser473), and Akt in the cytosolic fraction (20 µg protein) after treatment of AC2F cells with glucose (25 mM) for 1 h. β-actin was the loading control of the cytosolic fractions. Results are representative of three independent experiments. Bars in densitometry data represent means ± S.E., and significance was determined using one-factor ANOVA: $^{**}p < 0.01$ vs. Normal.

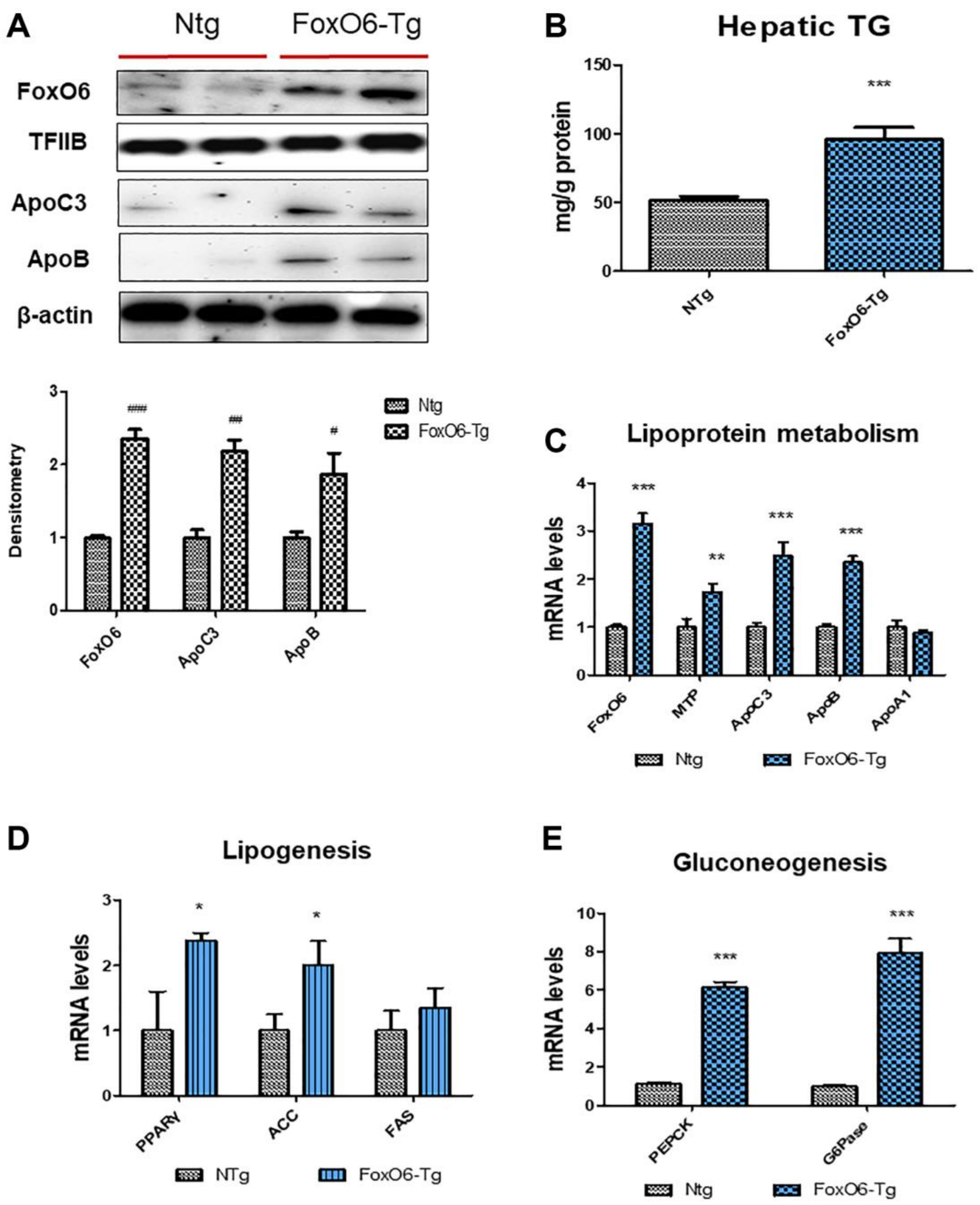


**Supplementary Figure 3. Regulation of the hepatic lipid accumulation in FoxO6-Tg mice.** (**A**) Western blotting was performed to examine the protein levels of FoxO6, ApoC3, and ApoB in the livers of FoxO6-Tg mice. TFIIB was the loading control of the nuclear fractions, whereas β-actin was the loading control of the cytosolic fractions. Bars in the densitometry data represent the mean ± S.E., and significance was determined using one-factor ANOVA: $^{\#}p < 0.05$, $^{\#\#}p < 0.01$, $^{\#\#\#}p < 0.001$ vs. Ntg. (**B**) Hepatic TG in FoxO6-Tg mice. Results of one-factor ANOVA $^{***}p < 0.001$ vs. WT littermates. (**C**) Real-time PCR analyses were performed for measuring the mRNA levels of FoxO6, MTP, ApoC3, ApoB, and ApoA1. Results of one-factor ANOVA: $^{**}p < 0.01$, $^{***}p < 0.001$ vs. WT littermates. (**D**) Real-time PCR analyses were performed for measuring the mRNA levels of PPARγ, ACC, and FAS. Results of one-factor ANOVA: $^{*}p < 0.05$ vs. WT littermates. (**E**) Real-time PCR analyses were performed for measuring the mRNA levels of PEPCK and G6Pase. Results of one-factor ANOVA: $^{*}p < 0.05$, $^{**}p < 0.01$ vs. WT littermates.
